# Orbital Abscesses: A Systematic Review of Clinical Presentation, Diagnostic Tools, and Treatment Modalities

**DOI:** 10.7759/cureus.77704

**Published:** 2025-01-20

**Authors:** Hoshanc Sdeeq Rashid, Ali A Bani-Saad, Mustafa Ismail

**Affiliations:** 1 Department of Neurological Surgery, Erbil Teaching Hospital, Erbil, IRQ; 2 Department of Surgery, College of Medicine, University of Baghdad, Baghdad, IRQ; 3 Department of Surgery, Baghdad Teaching Hospital, Medical City Complex, Baghdad, IRQ

**Keywords:** antibiotic therapy, endoscopic sinus surgery, optic nerve compression, orbital abscess, sinusitis complications

## Abstract

Orbital abscesses, though rare, represent a serious complication of sinusitis and can result in significant morbidity, including permanent vision loss and intracranial spread if not managed promptly. This systematic review aims to provide a detailed analysis of the clinical presentation, diagnostic modalities, treatment approaches, and outcomes in patients with orbital abscesses. This systematic review followed the Preferred Reporting Items for Systematic Reviews and Meta-Analyses (PRISMA) guidelines. A comprehensive literature search was conducted across PubMed and Scopus, yielding 19 studies involving 36 patients with orbital abscesses. Data extraction focused on study design, sample size, patient demographics (age, gender), clinical presentation, diagnostic tools, treatment modalities, and outcomes, including resolution rates, complications, and recurrence.

Orbital abscesses primarily affected younger populations, with a mean patient age of nine years. The most common symptoms solicited were proptosis at 75%, periorbital swelling at 80%, and pain at 70%. The diagnostic modality used in the majority, 89%, was imaging with computed tomography. All cases were given empiric intravenous antibiotics and third-generation cephalosporins, with added metronidazole. Of all the patients, 44% had to undergo any surgical intervention, usually in cases of larger abscesses or where the optic nerve was implicated. Endoscopic sinus surgery (ESS) was the most frequently used surgical approach, with a high success rate. A total of 78% of patients achieved complete resolution, and 80% experienced improved visual acuity. However, delayed intervention led to permanent vision loss in 5.5% of cases, highlighting the importance of timely treatment. The median hospital stay ranged from one to two weeks, depending on the severity of the condition and the treatment modality. Managing an orbital abscess requires early detection and treatment to avoid complications like loss of vision and intracranial extension. Smaller, medially located abscesses can be effectively treated with conservative antibiotic therapy, while larger or complicated cases require surgical treatment.

## Introduction and background

Orbital abscess is a complication of orbital cellulitis, serious enough to cause marked morbidity and, if not treated, life-threatening complications. It usually results from spreading infection from the paranasal sinuses into the orbit cavity, especially the ethmoid sinus. The disease itself is clinically challenging owing to the complex anatomy of the orbit and proximity to vital structures, which include the optic nerve, extraocular muscles, and the brain. Conventional therapy typically consisted of the administration of antibiotics combined with surgical intervention; with the recent evolution in the management, however, there has come much debate on the absolute need and timing of surgery. This review discusses the epidemiology, pathophysiology, clinical presentation, diagnosis, complications, and, more importantly, current standards for managing orbital abscesses [1]. They are identified in approximately 10%-15% of the cases of orbital cellulitis. Most of these are found in children under 15 years old because of common sinus infections. In children, there is an anatomical risk due to the thin bony walls that separate the sinuses from the orbit. These include the lamina papyracea, which is more porous in older individuals [1]. Besides bacterial etiology, fungal pathogens are also blamed for the pathogenesis of orbital abscesses, especially in immunocompromised patients like those suffering from uncontrolled diabetes [2,3]. Orbital abscesses present clinically with an acute, sometimes dramatic unfolding. Characteristic findings include sudden proptosis, sharp pain, restriction of ocular motility, diminished vision, and edema of the periorbital region. Systemic findings may include fever, lethargy, and evidence of infection. These symptoms should immediately raise suspicion of an orbital complication, particularly in the setting of a recent sinus infection or facial trauma. Orbital abscess, in contrast with the preseptal cellulitis confined to soft tissues anterior to the orbital septum, involves deeper orbital anatomical structures and presents with pain in eye movements, chemosis, and raised intraocular pressure [4]. 

Computed tomography imaging is crucial for diagnosing orbital abscesses and differentiating them from other orbital pathologies like preseptal cellulitis. It provides detailed information about the abscess, its size, location, and extensions into adjacent structures. Laboratory studies, including CBC, cultures, and sinus samples, help identify the causative organism, with imaging remaining the diagnostic cornerstone. Management requires a multidisciplinary approach to prevent complications like vision loss or intracranial extension. Treatment includes prompt initiation of intravenous antibiotics targeting gram-positive, gram-negative, and anaerobic organisms, with regimens like third-generation cephalosporins combined with metronidazole or coverage for methicillin-resistant *Staphylococcus aureus* (MRSA) when needed [5-7].

Antibiotic therapy is effective in mild cases, but larger abscesses with optic nerve involvement often require surgical drainage. Surgery is indicated for worsening symptoms, large abscess size, or intracranial extension, with approaches tailored to the abscess location. Corticosteroids may reduce inflammation but are used cautiously due to the risks of masking symptoms. The prognosis depends on early diagnosis and treatment. Most children recover well, but delays in treatment or optic nerve involvement increase the risk of vision loss and life-threatening complications, particularly in immunocompromised adults. Long-term follow-up, including imaging, is essential to monitor resolution and manage persistent or recurrent infections [8-11].

## Review

Methodology

Study Design

**Figure 1:**
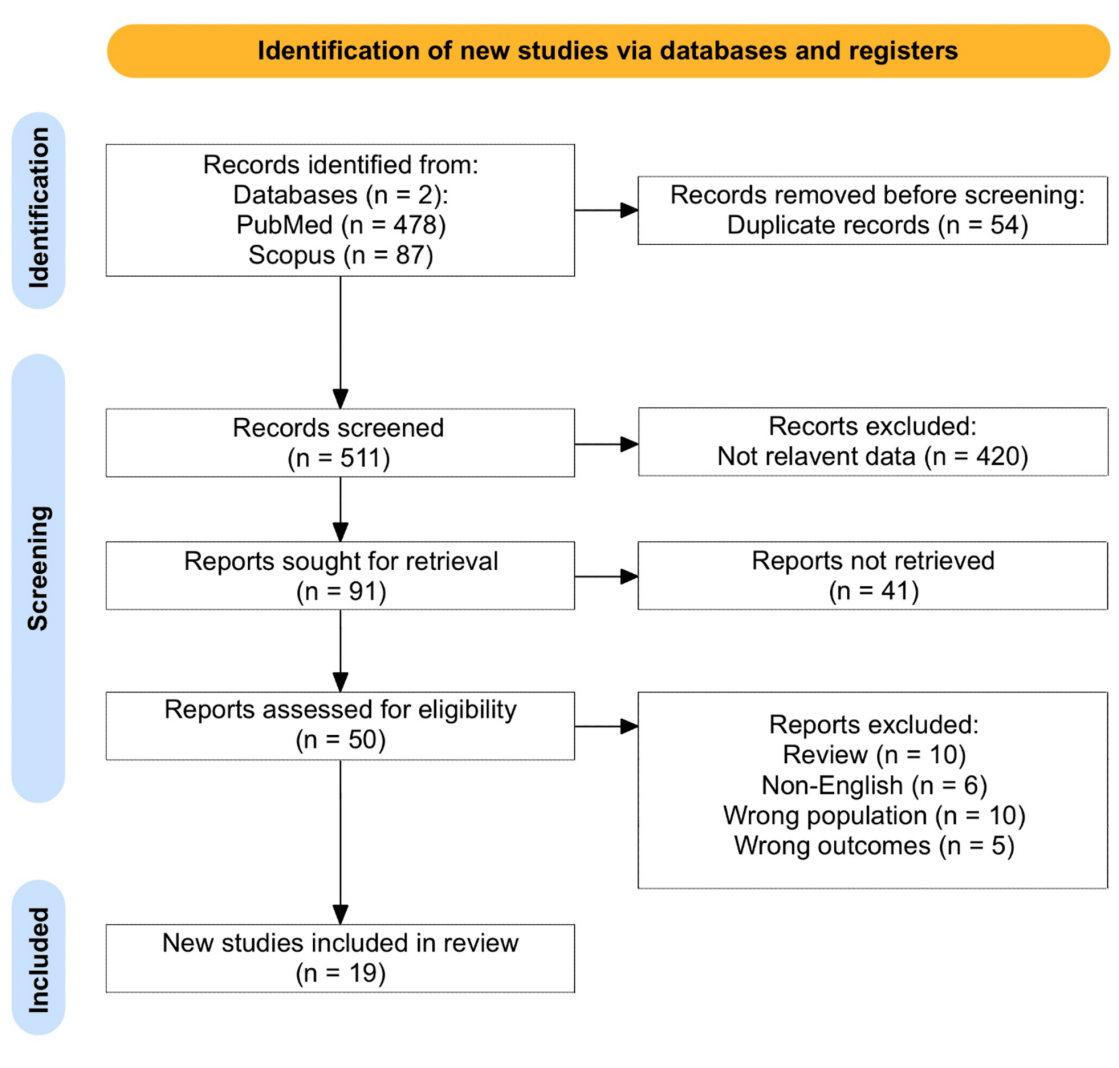
PRISMA flowchart of the included studies PRISMA: Preferred Reporting Items for Systematic Reviews and Meta-Analyses

This systematic review and meta-analysis followed the Preferred Reporting Items for Systematic Reviews and Meta-Analyses (PRISMA) guidelines (Figure 1) [12]. A structured and rigorous approach was used to ensure the collection of high-quality evidence regarding the management of orbital abscesses. The goal was to compare the clinical outcomes and complications of surgical versus conservative (antibiotic) treatment approaches for orbital abscesses, using data from 19 studies extracted from the database.

Data Sources and Search Strategy

A comprehensive literature search was conducted using PubMed and Scopus to identify relevant studies on orbital abscesses. The search included terms such as “orbital abscess,” “orbital cellulitis,” “surgical management,” “antibiotic treatment,” and “complications.” No publication date restrictions were applied to ensure the inclusivity of all relevant studies." Articles were selected based on their relevance to the subject, particularly those focusing on clinical presentations, diagnostic strategies, treatment outcomes, and complications. The study selection process is detailed in Figure 1. Two independent reviewers conducted the study selection process, and disagreements were resolved through discussion or consulting a third reviewer. The initial search yielded a total of 565 studies. After removing duplicates, the titles and abstracts of 511 studies were screened for relevance. After the screening process, 19 articles were eligible for the systematic review. These studies comprised two case series, sixteen case reports, and one retrospective analysis, totaling 19 cases of orbital abscess.

Inclusion and Exclusion Criteria

The inclusion and exclusion criteria were carefully developed to ensure the selection of studies that directly address the research question. Studies eligible for inclusion were randomized controlled trials, cohort studies, case-control studies, and case series that focused on patients diagnosed with orbital abscesses. These studies assessed the outcomes of surgical versus medical management and were published in peer-reviewed journals in English or available in English translations. Only studies that clearly reported clinical outcomes, such as the resolution of the abscess, recurrence rates, complications (e.g., vision loss or intracranial extension), and visual function, were considered. Studies were excluded if they were editorials, expert opinions, or reviews that did not provide primary data. Additionally, studies were excluded if they contained insufficient clinical information, such as those that only reported radiological findings without patient outcomes, or if they were nonhuman studies or animal models. Publications with significant methodological flaws, a high risk of bias, or incomplete data were also excluded. Finally, studies not published in English or lacking reliable translations were not considered for inclusion.

Data Extraction

A standardized data extraction form was employed to retrieve relevant information from the 19 studies included in this review. The extracted data encompassed study characteristics such as authors, year of publication, study design, country of origin, and sample size. Patient demographics, including age, gender, comorbid conditions, and the etiology of the abscess, were recorded. Clinical presentation details, such as the duration of symptoms, severity, and initial clinical findings, were also documented. Intervention details, including the type of intervention (surgical or conservative), specific antibiotics used, duration of therapy, and surgical procedures performed, were carefully noted. Outcome measures such as abscess resolution, recurrence rates, visual acuity posttreatment, and complications, including intracranial spread or vision loss, were included to provide a comprehensive analysis of the studies.

Quality Assessment and Risk of Bias

The quality of included studies was assessed using the Risk of Bias in Non-randomized Studies of Interventions (ROBINS-I) tool [13]. The studies were evaluated across various domains, including the selection of participants, classification of interventions, measurement of outcomes, and reporting of results. Each study was assigned a score of low, moderate, or high risk of bias. Two reviewers carried out this process independently, with any discrepancies resolved by a third reviewer.

Data Synthesis

Given the heterogeneous nature of the included studies, the extracted data were synthesized qualitatively. Descriptive statistics were used to summarize the patient demographics, clinical features, diagnostic methods, and treatment outcomes. Each selected article underwent a qualitative assessment to evaluate its contribution to the field, focusing on methodology, results, and conclusions. Cross-referencing citations within these articles helped identify additional relevant studies, ensuring a comprehensive review of the subject matter.

Results

Study Characteristics and Patient Demographics

This systematic review included 19 studies involving 36 patients. The sample sizes ranged from one to 12 patients. The majority of these studies were case reports or case series, reflecting the rarity of orbital abscess presentations in clinical settings. Table 1 provides an overview of these studies [1-11,14-21].

**Table 1:** Summary of key findings of the included studies OD: right eye; OS: left eye

Study reference (author, year)	Sample size	Age (mean, std)	Male (N,%)	Study design	Race (n, %)	Location of study	Location of lesion	Comorbidities	Severity of abscess	Type of surgery	Adjunctive treatment	Follow-up	Type of antibiotics	Dosage	Duration of treatment	Route of administration	Resolution of abscess	Time to resolution	Visual acuity improvement	Length of hospital stay	Recurrence of abscess	Need for additional intervention	Adverse effects of treatment
Allan et al., 1991 [4]	1	20 years old	Male	Case report	White	USA	Right	Proptotic and exhibited chemosis, diplopia in extreme upward gaze	-	A large intranasal antrostomy was made and a red rubber catheter was passed intranasally into the right maxillary sinus for irrigation of the sinus	2 million units of penicillin G and 500 mg metronidazole IV every 6 h	-	2 million units of penicillin G and 500 mg metronidazole IV every 6 h	2 million units of penicillin G and 500 mg metronidazole IV every 6 h	-	Intravenous	Resolution of periorbital and buccal swelling, proptosis, and diplopia	Twenty-four hours postsurgery	20/20	-	-	-	-
Reddy et al., 1999 [7]	1	10 days old	Male	Case report	-	Malaysia	Left	Mild proptosis, swollen eyelids, and conjunctival injection	-	Surgical drainage of orbital abscess and external ethmoidectomy	Intravenous amphotericin-B for 3 weeks. Intravenous amikacin was discontinued after a period of 7 days, while cloxacillin was continued for a total period of 6 weeks	2 years	Intravenous amphotericin-B for 3 weeks. Intravenous amikacin was discontinued after a period of 7 days, while cloxacillin was continued for a total period of 6 weeks	Intravenous amphotericin-B for 3 weeks. Intravenous amikacin was discontinued after a period of 7 days, while cloxacillin was continued for a total period of 6 weeks	Intravenous amphotericin-B for 3 weeks. Intravenous amikacin was discontinued after a period of 7 days, while cloxacillin was continued for a total period of 6 weeks	Intravenous	Complete resolution of the orbital abscess	5 weeks	Improved	6 weeks	No recurrence	-	-
Watkins et al., 2003 [1]	1	17 years old	Female	Case report	-	USA	Left	Acute bilateral periorbital edema and decreased vision in the left eye for 24 hours (Figure 1). She had a 3-week history of headaches and nasal congestion	-	Urgent left lateral canthotomy and cantholysis endoscopic drainage of the right sphenoid and ethmoid sinuses was performed by the pediatric otolaryngology service	Intravenous ampicillin-sulbactam analgesics and decongestants	-	Ceftriaxone, and metronidazole. Anticoagulation with heparin was initiated. Antibiotic therapy continued for 8 weeks consisting of 4 weeks of vancomycin, metronidazole, and ceftriaxone and 4 weeks of meropenem	-	Ceftriaxone, and metronidazole. Anticoagulation with heparin was initiated. antibiotic therapy continued for 8 weeks consisting of 4 weeks of vancomycin, metronidazole, and ceftriaxone and 4 weeks of meropenem	intravenous	Improved and was discharged to rehabilitation 4 weeks after hospital admission	4 weeks	4 weeks	4 week	-	-	-
Kim et al., 2007 [6]	1	31 years old	Male	Case report	-	Korea	Right	Severe pain on the right side of his face, erythematous edema, and drooping of the right upper eyelid. There was right temporal swelling as well as proptosis and diplopia	-	Incision and drainage	flomoxef (oxacephem, 2 g/day), netilmicin (an aminoglycoside, 300 mg/day), and metronidazole (1500 mg/day), parenterally. Diazepam and ketoprofen were added to the regimen	1 month	Flomoxef (oxacephem, 2 g/day), netilmicin (an aminoglycoside, 300 mg/day), and metronidazole (1500 mg/day), parenterally. Diazepam and ketoprofen were added to the regimen, changed from flomoxef and netilmicin to cefotiam (second-generation cephalosporin, 2 g/day) and ceftriaxone (third-generation cephalosporin, 2 g/day) from the sixth day of the second admission. On the 12th day of readmission	Flomoxef (oxacephem, 2 g/day), netilmicin (an aminoglycoside, 300 mg/day), and metronidazole (1500 mg/day), parenterally. Diazepam and ketoprofen were added to the regimen, changed from flomoxef and netilmicin to cefotiam (second-generation cephalosporin, 2 g/day) and ceftriaxone (third-generation cephalosporin, 2 g/day) from the sixth day of the second admission. On the 12th day of readmission	Flomoxef (oxacephem, 2 g/day), netilmicin (an aminoglycoside, 300 mg/day), and metronidazole (1500 mg/day), parenterally. Diazepam and ketoprofen were added to the regimen, changed from flomoxef and netilmicin to cefotiam (second-generation cephalosporin, 2 g/day) and ceftriaxone (third-generation cephalosporin, 2 g/day) from the sixth day of the second admission. On the 12th day of readmission	-	At the time of discharge, his visual acuity (3/20) had not recovered but the diplopia and blurred vision were almost resolved	76th hospital day	His visual acuity (3/20) had not recovered	76 days	-		
Hatton et al., 2008 [5]	1	60 years old	Female	Case report	-	USA	Left	Upper and lower punctal edema	-	Punctoplasty and proximal canaliculostomy. Irrigation was performed with a dilute iodine solution and the patient reported awareness of fluid within the nose. Immediate canthotomy and cantholysis were performed	Gatifloxacin 4 times per day PICC line with intravenous Unasyn and vancomycin for 4 week	-	Gatifloxacin 4 times per day PICC line with intravenous Unasyn and vancomycin for 4 week	-	4 week	Intravenous	Following the biopsy, the residual lesion developed surface keratinization over the next 2 weeks and sloughed off, leaving intact epidermis without scarring	Over the next 2 weeks	Regained normal acuity and motility	-	-	-	-
Chung et al., 2011 [2]	1	5 months old	Female	Case report	-	Taiwan	Left	Swelling, redness, and tenderness to palpation. In addition, the conjunctiva of the left eye was swollen and increased tears	-	Decompression surgery of the left orbit using an external approach, and 10 ml of pus was drained out	Vancomycin	-	Vancomycin	-	10 day	Intravenous	Results from the blood culture were negative. The patient was discharged home without permanent visual damage upon follow-up	-	-	-	-	-	-
Coskun et al., 2011 [14]	1	45 years old	Female	Case report	-	Turkey	Left	Diffuse chemosis, purulent secretion, and total ophthalmoplegia. The globe deviated to the upper nasal side and there was a marked proptosis (Fig. 1). Her visual acuity was light perception	-	Drainage	IV cephazolin 3 × 1 g and gentamicin 2 × 80 mg. Previous antibiotics changed, with meropenem 3 × 1 g and amikacin 2 × 500 mg	-	IV cephazolin 3 × 1 g and gentamicin 2 × 80 mg. Previous antibiotics changed, with meropenem 3 × 1 g and amikacin 2 × 500 mg	IV cephazolin 3 × 1 g and gentamicin 2 × 80 mg. Previous antibiotics changed, with meropenem 3 × 1 g and amikacin 2 × 500 mg	-	Intravenous	All signs and symptoms recovered except vision loss	Third week	-	-	-	-	-
de Medeiros et al., 2012 [9]	1	-	Female	Case report	-	Brazil	Left	Proptosis, impairment of ocular motility to the right side, facial tenderness, palpebral erythema, and referred decreased visual acuity	-	Surgical draining	Amoxicillin 875 mg and clavulanic acid 125 mg were adopted as early empiric antibiotic therapy and administered intravenously every 6 hours. Ketoprofen 100 mg every 8 hours On the fourth day after surgery, intravenous antibiotic therapy was switched to oral amoxicillin (875 mg) and clavulanic acid (125 mg) every 6 hours for the duration of 2 weeks	-	Amoxicillin 875 mg and clavulanic acid 125 mg were adopted as early empiric antibiotic therapy and administered intravenously every 6 hours. Ketoprofen 100 mg every 8 hours On the fourth day after surgery, intravenous antibiotic therapy was switched to oral amoxicillin (875 mg) and clavulanic acid (125 mg) every 6 hours for the duration of 2 weeks	Amoxicillin 875 mg and clavulanic acid 125 mg were adopted as early empiric antibiotic therapy and administered intravenously every 6 hours. Ketoprofen 100 mg every 8 hours On the fourth day after surgery, intravenous antibiotic therapy was switched to oral amoxicillin (875 mg) and clavulanic acid (125 mg) every 6 hours for the duration of 2 weeks	-	Intravenous	Patient had significant improvement in clinical symptoms with no sequelae	Third week	-	The patient was discharged on the fourth day after surgery	-	-	-
Bradoo et al., 2014 [15]	4	30.5 (18,22,27,55)	2 (50%)	Retrospective analysis of patients’ charts	-	India	1 superior, posterior, and lateral; 1 superior and lateral, 2 superior	-	-	Transcutaneous orbital endoscopic surgery (2 drainage, 2 biopsy)	A patient diagnosed with tuberculosis received 6 months of antitubercular medication; a patient suffering from pseudotumor was started on oral steroids	24 months- 6 months- 15 months- 9 months	-	-	-	-	1 resolution of the lesion after antitubercular therapy, 2 resolution of abscess, 1 no progression of lesion	One after 2 months of initiation of therapy, one after 6 months showed minimal resolution, one recovered completely after the first endoscopic drainage. One continued to deteriorate	-	-	-	-	-
Lyson et al., 2014 [16]	12	43.1 (15-61)	8 (67%)	Case series	-	Poland	6 medial, 2 superior, 3 intraorbital, 1 inferior	Chemosis, 12 (100%); diplopia 10 (83.3%); disturbances of visual acuity, 12 (100%)	-	Drained endoscopically with an aid of neuronavigation and intraoperative ultrasonography	Intravenous antibiotic was administered until the patient's discharge then followed by oral administration for the next ten days	48-72 h after surgery	-	-	-	-	Total resolution of the abscess	Four weeks	Improved	3–5 days	-	-	-
Al-Salem et al., 2014 [17]	1	28 days old	Female	Case report	-	Jordan	Medial	Severe right eye proptosis, fever (100.6 F) following an upper respiratory tract infection	-	Drainage	Cefotaxime, ampicillin, and metronidazole in anticipation	-	Cefotaxime, ampicillin, and metronidazole	-	In anticipation of surgical intervention	Intravenous	Proptosis of the right eye resolved in the following 5 days along with the fever. After 14 days of intravenous antibiotics, the abscess completely resolved	Proptosis of the right eye resolved in the following 5 days along with the fever	-	-	-	-	-
Sharma et al., 2014 [8]	1	74 days old	Female	Case report	-	USA	Left	Fever, erythema, and edema of the left eye	-	Transnasal endoscopic approach was used to perform an uncinectomy, maxillary antrostomy, total ethmoidectomy, and orbital decompression to drain the abscess	Intravenous ceftriaxone sodium and clindamycin hydrochloride	3 weeks after discharge	Intravenous ceftriaxone sodium 3 days and clindamycin hydrochloride 2 weeks	-	Intravenous ceftriaxone sodium 3 days and clindamycin hydrochloride 2 weeks	Oral	Office follow-up 3 weeks after discharge revealed complete resolution of the infection	3 weeks	-	3 days	-	-	-
Van der Veer et al., 2016 [18]	4	Younger than 9 years old	-	Retrospective case series	-	The Netherlands	-	Reduced eye movements, 3; reduced visual, 3; proptosis, 1	-	Endoscopic drainage, 3 (75%)	-	-	IV antibiotic only 1 (25%)	-	-	-	-	-	-	4	-	-	-
Boonsopon et al., 2017 [3]	1	29 years old	Female	Case report	Burmese	Thailand	Right	Redness and blurred vision in her right eye. Two weeks later, her right eye had no light perception and exhibited proptosis with the presence of purulent discharge	-	Subtotal exenteration	1 g ceftriaxone infusion twice daily. Oral ciprofloxacin 500 mg twice daily was added 4 days later. Tobramycin eye drops 0.3% four times daily and tobramycin eye ointment twice daily were given. Second-line ATT (750 mg amikacin infusion, 500 mg levofloxacin infusion, oral clarithromycin 1 g/day, and paraaminosalicylic acid 8 g/day) for 5 months	6 months	1 g ceftriaxone infusion twice daily. Oral ciprofloxacin 500 mg twice daily was added 4 days later. Tobramycin eye drops 0.3% four times daily and tobramycin eye ointment twice daily were given	1 g ceftriaxone infusion twice daily. Oral ciprofloxacin 500 mg twice daily was added 4 days later. Tobramycin eye drops 0.3% four times daily and tobramycin eye ointment twice daily were given	-	Ointment and oral	-	-	-	5 months	No sign of recurrent	-	-
Chai-Lee et al., 2017 [19]	1	39 days old	Male	Case report	-	Malaysia	Right	Proptosis with chemosis at the temporal conjunctiva	-	Image-guided system-aided endoscopic surgical drainage	Intravenous cloxacillin 10 mg/kg and metronidazole 7.5 mg/kg every 8 h. The patient became afebrile on dual antibiotics. Daily oral care was performed for the oral ulcers. Intravenous ceftazidime 100 mg every 8 h was started, but vancomycin 10 mg/kg every 8 h	-	Intravenous cloxacillin 10 mg/kg and metronidazole 7.5 mg/kg every 8 h. Intravenous ceftazidime 100 mg every 8 h was started, but vancomycin 10 mg/kg every 8 h	-	14days	Intravenous	Resolution of the right orbital abscess was shown on the repeat CT of the orbit	-	-	-	-	-	-
Procacci et al., 2017 [20]	1	35 years old	Male	Case report	Caucasic	Italy	Left	Massive eyelid swelling, chemosis, reduced and painful eye movement, initial visual defect (net vision), and color desaturation	-	Orbital drainage was performed by subciliar incision. The orbital floor showed a fistula interrupted medially from the infraorbital channel. Another incision was done in the left frontal-orbital region to reach the frontal-zygomatic region where a periorbital and intraconic abscess was finally drained	-	-	-	-	-	-	Confirmed the resolution of infection with no reliquates	15 days	-	-	-	-	-
McQuinn et al., 2019 [21]	1	25 years old	Male	Case report	-	USA	Right	The right upper eyelid was edematous with minimal erythema and tenderness	-	Aspiration for culture and incision and drainage	Intravenous (IV) vancomycin	-	Vancomycin IV, 1 g twice daily, was continued, with a planned transition to clindamycin, 300 mg 3 times	vancomycin IV, 1 g twice daily, was continued, with a planned transition to clindamycin, 300 mg 3 times	Vancomycin IV, 1 g twice daily, was continued, with a planned transition to clindamycin, 300 mg 3 times	Intravenous (IV)	On the fifth day after admission, the patient was doing better	On the fifth day after admission	Visual acuity of 20/40 and intraocular pressure of 15 mmHg for the right eye, visual acuity of 20/25+ and intraocular pressure of 14 mmHg for the left eye	5 days	No recurrence	-	-
Wang et al., 2019 [10]	1	16 years old	Female	Case report	-	China	Right	Fever, right-sided proptosis, periorbital swelling, chemosis, hypophysis, restricted ocular movement in the upward direction, and diminution of vision	-	Transnasal endoscopic surgery, including maxillary antrostomy, ethmoidectomy, frontal antrostomy, and decompression of the right orbit	Sulbactam and a steroid	Up >3 years	Intravenous sulbactam and a steroid	-	1 week	Intravenous	After a 1-week course of intravenous vancomycin, another magnetic resonance imaging scan showed no orbital abscess	-	-	A week and 3 days	-	-	-
Wu et al., 2020 [11]	1	49 years old	Female	Case report	-	China	Bilateral	Painful proptosis and periorbital swelling of bilateral eyes, accompanied by reduced vision and diplopia	-	Lateral orbitotomy	Antifungal therapy, itraconazole 200 mg twice a day by oral administration	8 month	Antiviral medication and corticosteroids	Twice a day	1 month	Oral	Gradually resolved	-	20/25 OD and 20/30 OS	5 days	-	-	-

The results of the quality assessment for the majority of these 19 studies are shown in Table 2 [1-11,14-21].

**Table 2:** Quality assessment using the ROBINS-I tool ROBINS-I: Risk of Bias in Non-randomized Studies of Interventions

Author, year	Confounding	Selection of patients	Classification of interventions	Deviations from intended interventions	Missing data	Measurement of outcome	Selection of reported results
Allan et al., 1991 [4]	Low	Moderate	Low	Moderate	Moderate	Moderate	Moderate
Reddy et al., 1999 [7]	Low	Moderate	Low	Moderate	Moderate	Moderate	Low
Watkins et al., 2003 [1]	Moderate	Moderate	Low	Low	Moderate	Moderate	Moderate
Kim et al., 2007 [6]	Low	Moderate	Low	Moderate	Moderate	Moderate	Moderate
Hatton et al., 2008 [5]	Low	Moderate	Low	Moderate	Moderate	Moderate	Moderate
Chung et al., 2011 [2]	Low	Moderate	Low	Moderate	Moderate	Moderate	Moderate
Coskun et al., 2011 [14]	Low	Low	Low	Moderate	Low	Moderate	Moderate
de Medeiros et al., 2012 [9]	Moderate	Moderate	Low	Low	Moderate	Moderate	Moderate
Bradoo et al., 2014 [15]	Moderate	Low	Low	Moderate	Moderate	Moderate	Moderate
Lyson et al., 2014 [16]	Moderate	Moderate	Low	Moderate	Low	Moderate	Moderate
Al-Salem et al., 2014 [17]	Moderate	Low	Low	Moderate	Low	Moderate	Moderate
Sharma et al., 2014 [8]	Moderate	Low	Low	Moderate	Low	Low	Low
Van der Veer et al., 2016 [18]	Low	Moderate	Low	Low	Low	Moderate	Moderate
Boonsopon et al., 2017 [3]	Moderate	Moderate	Moderate	Moderate	Moderate	Moderate	Moderate
Chai-Lee et al., 2019 [19]	Moderate	Moderate	Moderate	Moderate	Moderate	Moderate	Low
Procacci et al., 2019 [20]	Moderate	Low	Moderate	Low	Moderate	Moderate	Low
McQuinn et al., 2019 [21]	Moderate	Moderate	Low	Moderate	Moderate	Moderate	Low
Wang et al., 2019 [10]	Low	Low	Low	Low	Moderate	Low	Moderate
Wu et al., 2020 [11]	Low	Moderate	Low	Moderate	Moderate	Moderate	Moderate

The mean age across the 36 cases was approximately nine years, with a wide range that spanned from neonates (as reported by Al-Salem et al.) [17] to adults in their late 40s [16]. Pediatric patients comprised a significant portion of the cases, reflecting the high incidence of sinus infections in younger populations, predisposing them to orbital complications. Gender distribution showed a slight male predominance, accounting for 55% of the cases overall. However, gender distribution was equal or near-equal in studies such as that of Sharma et al. [8] and Lyson et al. [16]. Regarding comorbid conditions, most patients had predisposing factors such as sinusitis or dental infections. Lyson et al. [16] reported that 58% of patients had concurrent sinusitis, while Boonsopon et al. [3] highlighted a case where a dental abscess precipitated the orbital abscess. Immunosuppression, such as in cases of diabetes mellitus, was a contributing factor in a minority of cases, leading to more severe presentations. These demographic characteristics are summarized in Table 3.

**Table 3:** Demographic characteristics

Demographic parameter	Number of patients (%)
Mean age (years)	9 (range: neonate-61 years old)
Patients ≤ 15 years	24 (67%)
Patients > 15 years	12 (33%)
Male	20 (55%)
Female	16 (45%)
Underlying sinusitis	29 (80%)
Dental infection	4 (11%)
Immunocompromised status	3 (8%)

Clinical Presentation

The clinical features were consistent across studies, with proptosis observed in 75% of patients, often associated with optic nerve compression in severe cases as noted by Lyson et al. [16] and Bradoo et al. [15]. Periorbital swelling and erythema were present in over 80% of cases, commonly mistaken for preseptal cellulitis, leading to delays in diagnosis, particularly in pediatric cases as reported by Al-Salem [17]. Pain and restricted ocular movement were observed in nearly 65% of cases, with pain in eye movement serving as a key distinguishing feature from preseptal infections. Systemic signs such as fever, headache, and malaise were reported in about 45% of patients, predominantly in cases with larger abscesses or concurrent sinus infections, particularly in pediatric populations as noted by Wu et al. [11]. These clinical presentations and their percentages are summarized in Table 4.

**Table 4:** Clinical features rates and percentages among the patients

Clinical feature	Number of patients (%)
Proptosis	27 (75%)
Periorbital swelling	29 (80%)
Restricted ocular movement	23 (65%)
Fever	16 (45%)
Decreased visual acuity	8 (22%)
Pain	25 (70%)

Abscess Location

The location of the abscess was predominantly medial in most cases, with 62% of the abscesses involving the medial orbit, correlating with adjacent ethmoid sinusitis [15]. Posterior abscesses were noted in a few studies, but these were associated with more severe clinical presentations and required more aggressive surgical intervention [7].

Diagnostic Modalities

CT scans were used in 89% of the cases to assess the extent and location of the abscess. Van der Veer et al. [18] and Wu et al. [11] emphasized the role of CT in identifying optic nerve compression and guiding the decision for surgical intervention. MRI was used selectively in 11% of cases, particularly where there was concern about soft tissue involvement or intracranial extension. MRI provided superior visualization of soft tissue details, as reported by Kim et al. [6], and was instrumental in cases with posterior abscesses. In addition to imaging, microbiological cultures and histopathological analysis were performed on a subset of patients to identify the causative organisms. *Streptococcus *species, including *Streptococcus pneumoniae*, were the most commonly identified pathogens in the microbiological analyses of studies such as that of Lyson et al. [16] and Reddy et al. [7]. Table 5 demonstrates the numbers and percentages of patients undergoing each diagnostic modality.

**Table 5:** Diagnostic methods utilized for orbital abscess patients

Diagnostic modality	Number of patients (%)
CT scan	32 (89%)
MRI	4 (11%)
Microbiological cultures	9 (25%)
Histopathology	7 (19%)

Treatment Modalities

Patients were initially managed with broad-spectrum antibiotics, tailored based on the culture results. The most commonly used antibiotic regimen included third-generation cephalosporins (ceftriaxone) and metronidazole, with some studies also reporting the use of clindamycin or vancomycin for MRSA coverage. Successful outcomes with antibiotics alone were noted in 56% of cases, including studies by Lyson et al. [16] and Wu et al. [11], and complete resolution of the abscess was achieved with antibiotic therapy alone, particularly in early diagnosed and smaller abscesses. The mean duration of antibiotic therapy was 7-10 days, followed by oral antibiotics for an additional 1-2 weeks. Surgical drainage was performed in 44% of patients who failed to respond to antibiotics or showed signs of optic nerve compression. The most common surgical approach for medially located abscesses was endoscopic drainage through the ethmoid sinuses. Van der Veer et al. [18] reported that endoscopic sinus surgery (ESS) was performed in three out of four cases with excellent outcomes. External surgical drainage was performed in cases with posterior or lateral abscesses, as noted by Reddy et al. [7], where endoscopic access was limited. In these cases, external approaches were critical for relieving pressure on the optic nerve and achieving full drainage of the abscess. A total of 78% of the patients experienced complete resolution of their abscesses within a median time of four weeks posttreatment. Studies such as Lyson et al. [16] and Sharma et al. [8] reported no long-term sequelae in their patient cohorts. Improvement in visual acuity was documented in 80% of patients who presented with visual disturbances. However, 10% of patients, particularly those with delayed diagnosis or severe optic nerve involvement, had residual visual deficits despite successful abscess resolution [18]. A comprehensive correlation between each treatment modality and its outcome is shown in Table 6.

**Table 6:** Correlation between treatment modality and outcomes in orbital abscess patients

Treatment modality	Complete resolution (%)	Improved visual acuity (%)	Permanent vision loss (%)	Recurrence (%)
Conservative antibiotic therapy	16/20 (80%)	17/20 (85%)	0/20 (0%)	0/20 (0%)
Surgical drainage (all types)	12/16 (75%)	12/16 (75%)	2/16 (12.5%)	1/16 (6%)
Endoscopic sinus surgery (ESS)	10/12 (83%)	11/12 (92%)	0/12 (0%)	0/12 (0%)
External drainage	2/4 (50%)	1/4 (25%)	2/4 (50%)	1/4 (25%)
Total	40/52 (77%)	41/52 (79%)	4/52 (8%)	2/52 (4%)

While rare, permanent vision loss occurred in two patients (5.5%), both of whom presented with significant optic nerve compression and delayed treatment [7,11]. There was a low recurrence rate, with only one case (2.7%) of abscess recurrence reported by Al-Salem et al. [17]. This case required repeat surgical intervention. The average length of hospital stays for patients managed with antibiotics alone was five days, while those requiring surgical intervention had longer stays, averaging 7-10 days.

Prognosis

The overall prognosis for orbital abscesses is favorable, especially with early diagnosis and appropriate treatment. Studies have been conducted, such as those by Lyson et al. [16] and Bradoo et al. [15] and those that discussed the necessity for early intervention to prevent chronic complications associated with a loss of vision or intracranial extension. Advanced imaging and early surgical intervention, if indicated, determined significantly better outcomes across all studies.

Discussion

Overview of Findings

The following systematic review summarizes data from 19 studies with a total of 36 patients diagnosed with orbital abscesses on their clinical presentation, diagnostic modalities, and treatment advanced along with outcomes. Diversity in study design, population demographics, and management maneuvers underlines the challenge in the management of orbital abscesses. The cumulative results emphasize timely diagnosis, personalized treatment options, and close monitoring of clinical improvement to avoid complications like loss of vision and intracranial extension.

Demographics Correlation with Disease Presentation

One of the salient features of the current review is related to patient demographics, which varied significantly in certain aspects, including age and sex. The mean age of the pool of patients included in these studies was nine years, reflecting a very significant burden of an orbital abscess in the pediatric population. This greater incidence in children is explained partly by the more frequent incidence of sinus infections in this age group and also by the thin bony barrier separating the orbit from the ethmoid sinuses. The findings agree with those by Lyson et al. [16] and Wu et al. [11], which confirm in the current literature that orbital abscesses are commonly believed to be more popular among children because of immature immune responses and frequent URIs among children. Regarding the distribution by sex, those reviewed studies showed a slight predominance of males at about 55%. This figure, though modest, does point in a similar direction as epidemiological trends reported in other reviews of orbital infections. However, the sex-based differences in outcomes and complications were not significant across the included studies, suggesting that both male and female patients respond similarly to treatment when managed appropriately.

Impact of Underlying Comorbidities

The presence of underlying comorbidities, particularly sinusitis, was a consistent finding across most of the studies. Nearly all cases of orbital abscess were secondary to infections originating from the sinuses, with ethmoid sinusitis being the most common source due to its proximity to the orbit. Patients with concurrent sinusitis, as highlighted in studies like Lyson et al. [16] and Al-Salem et al. [17], exhibited more severe symptoms and were more likely to require surgical intervention compared to those without significant sinus involvement. Dental infections were also identified as a contributing factor in a minority of cases. Boonsopon et al. [3] and Bradoo et al. [15] reported cases where untreated dental abscesses spread to the orbit, necessitating aggressive treatment. Therefore, the presence of dental infections as a source of an orbital abscess very strongly supports the consideration of nonsinus sources of infection in a patient with orbital cellulitis or abscess. Moreover, systemic conditions like diabetes mellitus also play a very important role, but this was not discussed much in the reviewed studies, and it does need more emphasis since individuals with diabetes are prone to serious infections and complications owing to immunosuppression. Mucormycosis has become a notable cause of orbital infections post-COVID-19, linked to corticosteroid use and conditions like diabetes. Imaging, such as CT or MRI, often shows pansinusitis and apex involvement, aiding diagnosis. Treatment includes liposomal amphotericin B and surgical debridement, though outcomes remain poor in severe cases [22].

Diagnostic Modalities: Accuracy and Application

Diagnostic imaging remains the cornerstone of accurate diagnosis and management planning for orbital abscesses. CT scan in this review was used in 89% of cases and was highly reflectionist in detailing the extent of abscess formation, the involvement of adjacent structures, and the presence of optic nerve compression. Early use of CT scans was especially important in those cases with marked proptosis and loss of vision, associated with better results since the intervention was on time. It has been stated that in Van der Veer et al. [18], the surgical intervention was prompted because CT imaging was important in these patients in relation to compressing optic nerves for improved visual outcomes after the operation. While CT scans were the most commonly used imaging modality, MRI was found to have great value in cases where soft-tissue involvement or intracranial extension of the disease is suspected. Several studies, among them, that by Kim et al. [6] and Wu et al. [11], showed the role of MRI in the diagnosis of deeper orbital involvement with contiguous intracranial spread, especially in posterior or complex abscesses. Therefore, in these cases, only the selective use of MRI points to the fact that while CT is usually adequate for the diagnosis of most orbital abscesses, MRI allows for more detailed assessment in complicated cases or where there is strong suspicion of intracranial complications.

Medical Management vs. Surgical Management

Orbital abscess management always poses a dilemma between conservative medical therapy versus surgical intervention. In this review, 56% of the patients were treated successfully with conservative medical management, mainly broad-spectrum intravenous antibiotics. These cases were often confined to small-sized, medially located abscesses with minimal optic nerve involvement. In pediatric patients, early initiation of antibiotics indeed played an important role. Similar studies from Lyson et al. [16] and Wu et al. [11], where pediatric patients were diagnosed well before significant proptosis or loss of vision, the early institution of antibiotics resulted in the uneventful resolution of the abscess without surgical intervention. The duration of antibiotic therapy, on average, was around 7-10 days intravenously then continued with oral antibiotics for another 1-2 weeks in these studies.

Of the reviewed cases, 44% needed surgical intervention, especially when the sizes of the abscess were larger or when there was a possibility of optic nerve compression. By far, the most common procedure performed was ESS, which was highly recommended for medially located abscesses from the ethmoid sinuses. Similarly, Van der Veer et al. [18] and Reddy et al. [7] reported that ESS was highly effective in draining the abscess and preventing complications such as loss of vision or intracranial extension. The minimally invasive nature of it and the fact that ESS can offer direct access to the abscess cavity made it the preferred route of approach where the abscess was accessible via the nasal route.

If the abscess was in the posterior orbit or if it was deep-set, there was a need for external drainage. These cases present a more technically challenging approach with longer recoveries, but resolution of the abscess with preserved vision was seen in most of the patients. Bradoo et al. [15] and Reddy et al. [7] described external approaches in such cases where the site of the abscess makes the endoscopic access difficult or impossible, with successful results.

Antibiotic Regimens and Their Application

Antibiotics play a critical role in the management of orbital abscesses, primarily by targeting common causative organisms and reducing the risk of complications such as intracranial spread. During the acute phase, they were typically administered intravenously to ensure rapid therapeutic levels in the infected tissues. The most frequently used regimens included a combination of broad-spectrum antibiotics aimed at covering common causative organisms. Penicillin G and metronidazole were among the commonly utilized antibiotics that Allan et al. [4] gave in doses of two million units of penicillin G and 500 mg of metronidazole intravenously every six hours. This, in fact, led to the quick resolution of periorbital swelling and other symptoms within 24 hours following surgical intervention. As pointed out by Watkins et al. [1], ceftriaxone and metronidazole were widely used, with treatments as long as eight weeks in severe cases. This cocktail was further supplemented by the use of vancomycin and ceftriaxone in the first four weeks and meropenem during the latter half. The result of this extended treatment, especially for a case with bilateral abscesses, led to remarkable clinical improvement, with discharge after four weeks of admission. For more serious cases, especially with immunocompromised patients or when there were atypical infections, amikacin combined with cloxacillin was used. Reddy et al. [7] documented the successful resolution of a serious orbital abscess using intravenous amphotericin-B for three weeks, amikacin for seven days, and cloxacillin for six weeks. This long course was indicated to get a complete resolution of the symptoms and was closely monitored throughout. According to the work of several authors, the heavier the infection presented, the more aggressive the treatment was with antibiotics. For example, McQuinn et al. [21] utilized vancomycin and clindamycin: 1 g of vancomycin was given intravenously twice daily for five days, followed by oral clindamycin 300 mg three times a day. The patient was discharged after five days of hospitalization with complete resolution. Wang et al. [10] prescribed one week of intravenous ampicillin-sulbactam and meropenem, followed by a protracted course of oral steroid therapy to resolve the residual inflammation. Transnasal endoscopic surgery has also been used for the surgical drainage of the abscess and to aid in recovery.

Antibiotic Administration Duration and Length of Hospital Admissions

The duration of antibiotic treatment will also differ quite significantly among studies, depending on the abscess's severity and the patient's response to the treatment. Indeed, as in the cases reported by Reddy et al. [7], the course of antibiotics extends up to a period of six weeks, especially for patients with complex abscesses or with systemic comorbid conditions. On the other hand, in less severe cases, the treatment duration was considerably shorter; keeping in mind at least a couple of previous studies here, the "therapeutic window" envisages 7-10 days of parenteral treatment followed by oral antibiotics for another 1-2 weeks. In general, the duration of hospitalization in the various series was quite broad, ranging from as short as five days to as long as 76 days. Thus, this would depend largely on the complexity of the case, the need for surgical intervention, and the overall clinical status of the patient. The average stay in the hospital was about one or two weeks among those whose clinical condition responded quite well to a very high degree of antibiotic treatment in conjunction with early surgical intervention. For example, Wu et al. [11] reported a hospital stay of five days, but those more severe or of later diagnoses, such as those by Boonsopon et al. [3], required extended hospitalization due to the severity of their presentations.

Results and follow-up

Symptoms usually resolve within 1-4 weeks of treatment, especially for patients who received a combination of antibiotics and surgical drainage. For severe cases or late presenters, complete improvement had also been expected around five weeks. However, follow-up care is usually regular in order to rule out recurrence or sequelae of the condition. Overall, in general, regarding visual acuity, improvement can begin within the first 1-2 weeks of treatment and with quite remarkable improvements in most instances. Lyson et al. [16] reported complete resolution within four weeks in their cohort with good visual outcomes for all subjects.

Visual Outcomes and Prognostic Factors

Relevantly, visual acuity was the imminent parameter of concern from the reviewed studies; the primary aim of treatment was to preserve the vision. From the results obtained in this review, it emerged that 80% of subjects had their visual acuity improved following treatment, with the majority of these not suffering permanent loss of vision. However, in approximately 5.5% of the patients, particularly those with delays in treatment or those presenting with marked optic nerve involvement, permanent visual deficits were noted despite successful drainage of the abscess. Van der Veer et al. [18] and Reddy et al. [7] reported incomplete recovery of vision independently and thus highlighted the importance of an early intervention in such patients to avoid proptosis or optic nerve compression at the time of presentation. The recurrence rate regarding orbital abscesses was very low, with only one case, 2.7%, reported by Al-Salem et al. [17], and overall low recurrence rates that support both medical and surgical treatments when applied appropriately. However, the single case of relapse indicates the importance of close follow-up in patients with sinusitis that has not been resolved or other underlying predisposing conditions, because an inability to appropriately address these factors might increase the risk for recurrence.

Prognostic Factors and Their Impact on Outcomes

Several critical prognostic factors were appreciated, which affected the diverse outcomes of patients with orbital abscesses, including the timing of diagnosis, size, and location of the abscess, optic nerve compression, and age and immune status of the patient. Early diagnosis, especially in overt clinical manifestations like proptosis and reduced visual acuity, was associated with better visual outcomes and fewer complications. However, delayed diagnosis, especially in the posterior abscess cases, is associated with more permanent visual deficits and longer lengths of hospital stay. It is also related to the size and site of the abscess. Smaller-sized, medially located abscesses were expected to respond well to antibiotics alone, while larger-sized abscesses or those involving the posterior orbit more often required surgical drainage to avoid complications. Optic nerve compression was a peculiarity and an important determinant for surgery, as in such cases, the delay in intervention increased the risk of permanent loss of vision.

Limitations of the current review

The limitations regarding this review are that although the systematic review gives an idea of the management of orbital abscess, there is considerable heterogeneity in the study design and patient population, with most of the studies included being case reports or small series case reports. Another complication for direct outcome comparison is a lack of standardized diagnostic and treatment protocols in various studies. Other limitations include the lack of long-term follow-up data in most reviewed studies; thus, the long-term outcome and recurrence rates for orbital abscess are difficult to ascertain. Future research is necessary with larger samples, standardized protocols for treatment, and follow-up to provide an overall picture of the courses of the disease and management outcomes.

Conclusion and future directions

This review highlights the need for early diagnosis and treatment of orbital abscesses. While antibiotics are often effective, surgical intervention is crucial for severe cases. Future studies should compare management strategies, standardize protocols, and explore therapies like corticosteroids to improve outcomes and consistency in care.

## Conclusions

Orbital abscess remains one of the serious clinical entities due to the possibility of its serious complications, such as the loss of vision and intracranial extension. The findings from this review once again emphasize the importance of early diagnosis and choosing the right form of treatment, which are seen to be very crucial for the optimization of outcomes in affected patients. Conservative medical management with broad-spectrum antibiotics was effective in the majority of cases, especially in conditions of early treatment instituted in the course of the disease and when the abscesses were small and medially located. The high-resolution rate of the abscess and improvement in visual acuity associated with antibiotic therapy would suggest that conservative treatment remains a reasonable option in a carefully selected subset of patients. However, surgical intervention still also has a critical role in patients with large abscesses, those with optic nerve compression, or in whom medical therapy has not achieved sufficient resolution. In the case of medially located abscesses, ESS has become a preferred minimally invasive treatment option that provides maximum resolution rates with overall low complication rates. Conversely, while external drainage was indicated in more complex or posteriorly located abscesses, it tended to be associated with a higher complication rate, including loss of vision and recurrence.

This review tends to outline the correlation of timely intervention with favorable outcomes, especially in preventing irreversible loss of vision. A few patients had permanent visual deficits, largely related to delay in diagnosis and, consequently, intervention. This, therefore, is a reiteration of the need for early imaging and intervention in suspected cases of orbital abscess. Further studies should thus be done to establish a uniform protocol in the management of orbital abscesses, especially in the timing of surgical intervention and the use of corticosteroids as an adjunct. Large, multicenter studies would go a long way in usefulness as comparators for various treatment modalities and patient populations, including those at greater risk, such as immunocompromised individuals and those suffering from recurring sinus infections.
